# Can the Pediatric Early Warning Score (PEWS) Predict Hospital Length of Stay?

**DOI:** 10.7759/cureus.11339

**Published:** 2020-11-05

**Authors:** Obeid M Shafi, Juan D Diego Rondon, Gagan Gulati

**Affiliations:** 1 Department of Pediatrics, Flushing Hospital Medical Center, New York, USA

**Keywords:** pediatric scoring systems, pediatric emergency, length of stay, observation units, pediatric early warning score (pews), hospital admission, healthcare administration

## Abstract

Background: Limited studies have evaluated the utility of scoring systems in the pediatric emergency department (PED) and no studies have evaluated their ability to predict hospital length of stay (LOS) and the usage of Observation units (OUs).

Objective: To evaluate the utility of the Pediatric Early Warning Score (PEWS) in predicting LOS in pediatric patients and thus anticipate admission to an OU versus the pediatric ward.

Methods: A retrospective study of pediatric inpatients (0 to 18 years) at an inner-city community hospital between January 2014 and December 2014. Patients with psychiatric illness, non-medical reasons for hospital stay, and those not discharged to ‘home’ were excluded. Demographic data, PEWS in the ED, and LOS for each patient were recorded and analyzed.

Results: A total of 719 patients were analyzed. PEWS range was 0 to 8. The mean LOS was 56.8 hours for patients with PEWS 0-1 compared to 62.7 hours for patients with PEWS ≥2 (p=0.02). There was a significant difference in PEWS for LOS ≤24 and ≤36 hours in comparison to those with LOS >24 hours and >36 hours, respectively (p<0.001). Overall, the PEWS correlated with LOS (r=0.11, p=0.002). Age correlated inversely with LOS (r=-0.16, p<0.001), without correlation to PEWS (r=-0.002, p= 0.96).

Conclusions: PEWS correlated weakly with LOS. A statistically significant lower PEWS was observed for patients who had short stays (both ≤24 and ≤36 hours) in comparison to those requiring longer inpatient care. Therefore, the PEWS is a useful tool to predict LOS and aid ED physicians to determine disposition, although further prospective studies in centers with OUs would better characterize its ability to suggest admission to an OU compared to the wards.

## Introduction

The emergency department (ED) is often the initial point of contact for patients seeking medical care. To provide high-quality medical services, the prompt identification of patients at risk of clinical deterioration and the provision of a certain level of medical care commensurate with the severity or acuity of illness is essential [1]. Even though a significant proportion of pediatric ED (PED) visits are of non-urgent nature, they have the potential to overburden an already congested health care system increasing the risk of adverse outcomes, impairing access, and possibly decreasing quality of care [2]. In terms of patient disposition, inappropriate hospital admissions can lead to increased costs and carry the risk for nosocomial infections. On the contrary, under-admissions can lead to a delay in diagnosis and treatment which can be life-threatening or cause increased morbidity [3]. Various pediatric scoring systems have been developed to provide an objective assessment of a patient’s clinical status based on physiologic parameters. One of the earliest and simplest scoring systems was the Pediatric Early Warning Score (PEWS) described by Monaghan [4,5]. Although the pediatric scoring system was developed to evaluate inpatients, its use has also been expanded to the PED [1,6-10].

There is a paucity of evidence evaluating predictive factors for hospital length of stay (LOS) in pediatric patients. Independent variables of sex, age category, specialism, risk of malnutrition, complications, need for home care and the involvement of other disciplines have been reported to be associated with LOS [11]. Since children with greater acuity of illness would logically require a prolonged hospital stay, our study aims to study the relationship between PEWS calculated in the ED and hospital LOS. Furthermore, pediatric observation units (POUs) are emerging as alternative sites of care for children and adolescents with select diagnoses, with the goals of reducing inpatient admissions, improving efficiency, improving patient safety, and controlling costs [12,13]. The use of Observation units (OU) was first conceptualized for adults, and subsequently, POUs have been set up [14,15]. Previous data have shown that pediatric patients are often hospitalized for brief durations, with nearly one-third of pediatric admissions hospitalized for one night or less [16-18]. POUs are ideal for monitoring serial physical examinations, awaiting consultations, administering short courses of treatment, and enabling providers to better decide if hospitalization is warranted in cases where the disposition is unclear after an initial assessment [12,13,15]. OU can be a designated, dedicated area, distinct from the ED and inpatient area or it may be a virtual OU, where certain beds within the ED or hospital ward are allocated. In either case, the main purpose of the designation is an administrative classification for purposes of reimbursement. OUs can have a great financial impact, as they have the potential of converting non-reimbursable hospital admissions into profitable observation stays [15,19].

Limited studies have evaluated the utility of scoring systems in the pediatric ED and no studies have evaluated their ability to predict hospital LOS and the usage of OU. We postulate that an objective pediatric scoring system could predict the LOS in pediatric inpatients and thus anticipate admission to an OU versus the ward.

## Materials and methods

This was a retrospective analysis of data from consecutive patients admitted as inpatients from the PED of a community hospital in New York City from January 01, 2014 to December 31, 2014. The data from all patients between 0 to 18 years of age was retrieved, anonymized and reviewed. Patients admitted specifically for inpatient psychiatric evaluation and/or treatment were excluded from our study, as these admissions were primarily transitory to prevent self-harm and pending transfer to a dedicated child psychiatric unit. Also, patients admitted for and/or whose discharge was delayed due to social reasons, and those whose discharge disposition was other than ‘home’ were excluded from our study. We collected demographic data including age, gender, ethnicity, and primary diagnosis for all patients included in the study. The diagnoses were subdivided based on a systems approach into respiratory, gastrointestinal, fever, neurological or miscellaneous. The PEWS was calculated retrospectively by one of the authors based on routinely collected clinical parameters entered in the electronic chart of the patient at initial assessment in the ED (Tables 1-2), and the LOS for each patient was recorded [4,9,10]. The PEWS was calculated based on five parameters: behavior, cardiovascular status, respiratory status, frequent nebulizer use, and persistent postsurgical vomiting. Data were analyzed using Microsoft Excel and GraphPad Prism software (GraphPad Software Inc., San Diego, CA), applying descriptive statistics, t-tests and Spearman correlation (r) analysis, with p<0.05 considered significant.

**Table 1:** Pediatric Early Warning Score (PEWS) *Score 2 extra for persistent vomiting following surgery or ¼ hourly nebulizers.

	0	1	2	3
Behavior	Playing/ appropriate	Sleeping	Irritable	Lethargic/confused or reduced response to pain
Cardiovascular	Pink or capillary refill 1-2 s	Pale or capillary refill 3 s	Gray or capillary refill 4 s or tachycardia of ≥20 bpm above normal rate	Gray and mottled or capillary refill ≥5 s or tachycardia ≥30 bpm above normal rate or bradycardia
Respiratory	Within normal parameters, no retractions or tracheal tug	Respiratory rate ≥10 breaths/min above normal parameters, using accessory muscles or 30%+ FIO2 or 3+ L/min	Respiratory rate ≥20 breaths/min above normal parameters, retractions, tracheal tug, or 40%+ FIO2 or 6+ L/min	RR 5 breaths/min below normal rate with retractions and/or grunting, or 50%+ FIO2 or 8+ L/min

**Table 2:** Normal vital sign ranges

	Heart Rate (beats per minute)	Respiratory Rate (breaths per minute)
Newborn (<31 days)	100-180	40-60
Infant (1 to 12 months)	100-180	35-40
Toddler (13 months to 3 years)	70-110	25-30
Preschool (4 to 6 years)	70-110	21-23
School age (7 to 12 years)	70-110	19-21
Adolescent (>12 years)	55-90	16-18

## Results

There were 737 inpatient admissions in the study period, and 18 patients were excluded from the study after chart review based on exclusion criteria. The final data from 719 inpatients were collected and analyzed.

**Figure 1:**
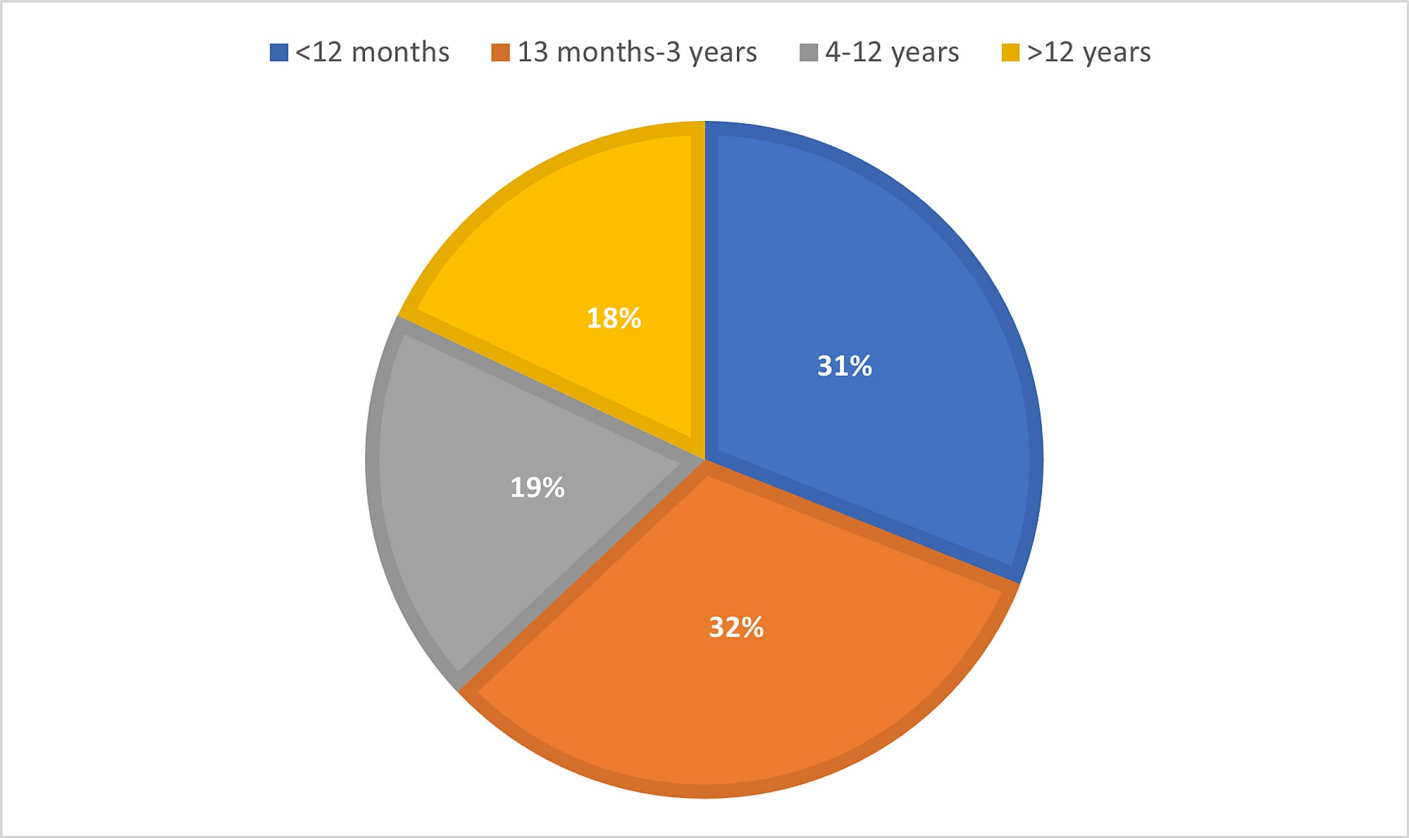
Age distribution of study population

The study population reflected a slight male preponderance (395/719, 55%), while the rest were female (324/719, 45%). Patients with a varied age distribution were part of the inpatients studied, with infants <12 months (221/719, 31%) and toddlers between 13 months-three years (231/719, 32%) being the most common, while the rest 19% (137/719) and 18% (130/719) were between 4-12 years and >12 years respectively (Figure 1).

The majority of our patients (460/719, 64%) identified as being of Latin American descent. The most frequent diagnosis was involving the respiratory system (pneumonia, asthma, bronchiolitis, etc.) (201/719, 28%), followed by fever (86/719,12%), and gastrointestinal illness (oral intolerance, abdominal pain, acute gastroenteritis, etc.) in 9% (65/719). 

**Figure 2:**
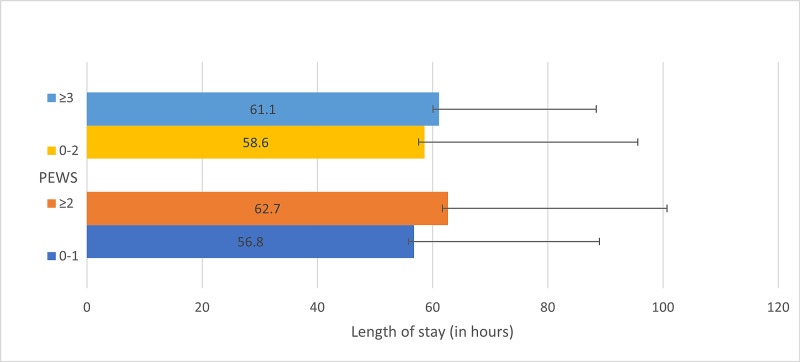
Length of stay (LOS) in patient groups with different Pediatric Early Warning Score (PEWS) cut-offs

The range of calculated PEWS (0 to 8) in our study was similar to that reported by Breslin et al. (0 to 7) in patients admitted to the acute care unit [9]. We compared the LOS for patient groups utilizing different PEWS cut-offs previously used to predict hospital admission and severity of illness [9,10]. The mean LOS was 58.6 hours (SD 37, n=535) and 61.1 hours (SD 27.3, n=184) in patients with PEWS of 0-2 and ≥3 respectively, with p-value of 0.39 (95% CI -8.35 to 3.33) when comparing the groups. Whereas, the mean LOS was 56.8 hours (SD 32.2, n=425) for patients with PEWS 0-1 compared to a LOS of 62.7 hours (SD 38, n=294) for patients with PEWS ≥2, with p=0.02 (95% CI -11.1 to -0.8) (Figure 2).

The mean LOS was 59 hours (Range 13 to 458 hours). The LOS was 24 hours or less in 60 patients, while 159 patients had LOS of 36 hours or less. There was a significant difference (p<0.001, 95% CI -1.17 to -0.35) for the calculated PEWS in patients with LOS≤24 hours (mean 0.58, SD 1.12, n=60) in comparison to those where the LOS>24 hours (mean 1.34, SD 1.59, n=659). Similarly, the PEWS score was significantly lower in patients with LOS≤36 hours (mean 0.85, SD 1.35, n=159) compared to LOS>36 hours (mean 1.4, SD 1.61, n=560) with p<0.001 and 95% CI -0.83 to -0.28. Overall, the PEWS correlated with LOS (r=0.11, 95% CI 0.04 to 0.19, p=0.002).

Patients with age equal and less than three years had a LOS greater than those aged 4 and above, by 9 hours on average. The age of the patients correlated inversely with the LOS (r=-0.16, 95% CI -0.2 to -0.08, p<0.001). However, the assigned PEWS did not have a significant correlation with the age of the patients (r= -0.002, 95% CI -0.07 to 0.07, p= 0.96).

## Discussion

The ED plays a pivotal role in the early and accurate identification of patients at risk for deterioration. Changes in physiological parameters have been demonstrated to occur hours prior to cardiac arrest in both children and adults [20]. The PEWS was developed to provide a means for standardized and reproducible identification of admitted pediatric patients at risk for deterioration in clinical status [4]. The PEWS described by Monaghan can be quickly performed, without age group restrictions and has been validated in a retrospective study of the inpatient floor setting at a pediatric hospital to identify patient deterioration [5]. The use of the PEWS has subsequently been expanded to the PED, and is associated with the level of care at ED disposition and could serve as a tool for prediction of ICU admission [1,6-10]. These scoring systems have not been studied in their ability to predict the hospital LOS.

The PEWS has been previously studied in its ability to guide hospital admissions at tertiary-care children's hospitals. Lillitos et al. [10] in their study proposed a PEWS cut-off of 3 or more for consideration of hospital admission and investigation for serious illness (Sensitivity: 32%, Specificity: 93%). In the same study, a lower cut-off with PEWS of 2 or more provided almost twice the sensitivity (62%) with a reduced specificity (68%) for hospital admission. The authors also suggested that a low PEWS does not exclude significant illness or the need for admission. Pertinently, the authors had automatically assigned a PEWS of 1 to every patient for ‘parental concern’; a practice that was not adopted in our study.

Breslin et al. [9] reported that although it was not possible to assign a single cut-off score to determine patient disposition due to the poor discriminatory ability of the PEWS, scores of 1 or greater at ED disposition had the greatest discriminatory ability in predicting need for admission albeit with doubtful clinical significance (Sensitivity: 63%, Specificity: 68%). In contrast, PEWS of 2 or more would identify a subgroup of patients with more than twice the probability of admission (Sensitivity: 44%, Specificity: 80%), at the expense of missing the majority of patients needing admission. Bradman et al. [7] also demonstrated that PEWS calculated at the time of ED triage had limited value to detect the need for admission in view of low sensitivity with PEWS of 2 or more having sensitivity of 37% and specificity of 88% in detecting the need for admission.

As our study included only admitted patients, it was not possible to directly calculate the discriminatory ability of PEWS in determining the need for admission. We compared the LOS in patients who would have been deemed to be ‘needing admission’ or at ‘risk for significant illness', based on PEWS cut-offs from previous studies [7,9,10]. A cut-off score of 2 or more at initial assessment in the ED provided a statistically significant difference (p=0.02) for LOS, with mean LOS of 56.8 hours for patients with PEWS 0-1 compared to a mean LOS of 62.7 hours for patients with PEWS ≥2. Comparing patient groups with PEWS of 0-2 and ≥3, the mean LOS was 58.6 hours and 61.1 hours respectively, with p-value of 0.39. Thus, using a PEWS cut-off of 2 or more resulted in a statistically significant difference for LOS in the patient groups, with an average difference of almost 7 hours.

We observed a weakly positive correlation between the calculated PEWS with the hospital LOS for patients (r=0.11, p=0.002). The calculated PEWS in patients with LOS≤24 hours and ≤36 hours was lower than those where the LOS of stay exceeded 24 hours and 36 hours respectively, suggesting that patients with shorter stays had lower PEWS at initial assessment in the ED. However, deciding the PEWS cut-off for observation versus admission would need further studies. We also observed the age of the patient to inversely correlate with the LOS in our study (r=-0.16, p<0.001), without correlation to the assigned PEWS. Age as an independent variable for LOS in pediatric patients has been previously reported by Tump et al [11].

A limitation of this study was that it was a retrospective study performed at a single-center. The PEWS was calculated at the first assessment in the ED, with the potential for unaccounted changes in physiological parameters over time. However, the calculation of PEWS at ED disposition has its own limitations as physiological parameters can be altered by treatments provided in the window from initial presentation to ED disposition, resulting in an inappropriately high/low PEWS [9]. In addition, although the severity of illness would logically be one of the most important factors dictating hospital LOS, various other factors could also play a vital role in pediatric patients, especially those with underlying complex medical problems. Other factors including the age of the patient (as observed in our study) and social factors have also been reported to play a role [11], although we tried to account for social factors by excluding such patients in our study. Another limitation of our study was the absence of an OU at our center, and further studies at centers with OUs could provide more insight into the relationship between PEWS and LOS, particularly in those patients requiring short stays.

## Conclusions

We report a statistical correlation (r=0.11) between PEWS calculated at initial assessment in the ED with LOS, suggesting the applicability of pediatric scoring systems at the PED of community hospitals. A significantly lower PEWS was observed for patients who had short stays (both at ≤24 and ≤36 hours) in comparison to those requiring longer inpatient care (p<0.001). Thus, PEWS is a useful tool and objective assessment to predict LOS. It could aid ED physicians to determine disposition, help healthcare managers to plan better and potentially cut costs, although further prospective studies in centers with OUs could better delineate its ability to suggest admission to an OU compared to the wards.
